# Hemispatial Neglect: Computer-Based Testing Allows More Sensitive Quantification of Attentional Disorders and Recovery and Might Lead to Better Evaluation of Rehabilitation

**DOI:** 10.3389/fnhum.2013.00162

**Published:** 2013-05-01

**Authors:** Mario Bonato, Leon Y. Deouell

**Affiliations:** ^1^Department of Experimental Psychology, Ghent UniversityGhent, Belgium; ^2^Department of Psychology, Edmond and Lily Safra Center for Brain Sciences, The Hebrew University of JerusalemJerusalem, Israel

Past studies aiming to test the effectiveness of rehabilitation techniques for hemispatial neglect have been often criticized for a number of methodological limitations, from non-random assignment to the groups, to absence of blind scoring (Cicerone et al., 2000; Cappa et al., 2005; Bowen and Lincoln, 2007; Paci et al., 2010; Teasell et al., 2011). While it seems that these shortcomings are being addressed by more recent studies, we here maintain that a major methodological improvement in studies of neglect rehabilitation might derive from the adoption of computer-based assessment, which has several advantages over the commonly used bed-side clinical or paper-and-pencil (PnP) tests. These more sensitive measures of neglect may provide a more accurate assessment of the effect of rehabilitation procedures, which may be missed with the currently employed classical measures of neglect, and may provide an indication for rehabilitation in patients who are currently not treated because of their normal performance on PnP tests.

Unfortunately, to our knowledge, there are very few rehabilitation studies utilizing such diagnostic tasks and they are mostly focused on rehabilitation of sustained attention (DeGutis and Van Vleet, 2010; Van Vleet and Degutis, 2013; see also Finke et al., 2012).

Paper-and-pencil tests are routinely adopted to measure patients’ performance after stroke. They are used in the acute phase to select the patients which will undergo rehabilitation, and in the chronic phase to monitor patients’ performance before, during, and after rehabilitation. PnP tests suffer however from various limitations which are particularly evident when the tests are repeatedly administered during recovery (see Deouell et al., 2005 for discussion). First, PnP tests typically do not change from one examination to the next, allowing for significant learning and compensatory strategies. Second, they are static, further allowing the implementation of compensatory strategies while not reflecting the dynamic character of the natural environment. These characteristics, coupled with the fact that only accuracy is measured, lead to early “normalization” of PnP scores, or a ceiling effect, when the patient may still demonstrate significant behavioral abnormalities in everyday life situations. Furthermore, in cancellation tests, a common type of PnP test, the tests are typically summarized into a single score, with no indication of performance variance which may be in itself a sensitive marker of the deficit (Anderson et al., 2000).

The sensitivity of some PnP tests may be increased by scoring measures that are sensitive to specific deficits. However, most of finer-grained approaches to PnP test scoring cannot be applied *a posteriori*, even when the raw tests are available [with the exception of the Center of Cancellation (Rorden and Karnath, 2010) for cancellation tasks]. For instance, execution time or start- and end-point require additional information to be registered by a trained examiner while performing the test (Manly et al., 2009; Buxbaum et al., 2012), which is not always feasible. Moreover, they provide only gross measures of performance with respect to the wealth of information potentially available through computer-based tests. The quantitative assessment of drawing tests is also problematic given the heterogeneity of potential errors (Seki and Ishiai, 1996), and paucity of normative data. Overall the sensitivity of the PnP tests in the post-acute and chronic phases cannot be considered satisfactory (Azouvi et al., 2002; Deouell et al., 2005; Hasegawa et al., 2011; Bonato, 2012). Thus, whereas PnP tests may be acceptable to assess neglect at the bed-side in the acute phase (Nijboer et al., in press), at later stages computer-based tasks provide more sensitive and informative assessment, allowing to detect contralesional impairments in performance even in patients who perform normally at PnP tests (Schendel and Robertson, 2002; Deouell et al., 2005; Erez et al., 2009; Bonato, 2012; van Kessel et al., 2013).

Compared to PnP tests, more sensitivity and flexibility is offered by computerized tests (Schendel and Robertson, 2002; List et al., 2008), which typically record much more information (e.g., accuracy and reaction time measures simultaneously). Stimuli may be presented in varying locations and times across trials, sessions, and sensory modalities, and repeated many times (Deouell et al., 2005; Bonato et al., 2010; Buxbaum et al., 2012; Van Vleet and Degutis, 2013). Various difficulty levels can be easily implemented and eventually combined with concurrent tasks to manipulate the load, and may be combined with other measures (e.g., eye movements, Van der Stigchel and Nijboer, 2010; touch screen recording, Rabuffetti et al., 2012). These features, along with the addition of RT measures, reduce the chances for ceiling effects and allow for quantitative, continuous measures, and even significance levels in single patients, including sensitive individual monitoring of performance changes through repeated assessments. Because of their unpredictable nature (presenting stimuli in random places, shapes, and times), the computerized tests are harder to learn, and to develop compensatory strategies for. They are thus more suitable for test-retest designs, which are a *sine qua non* in rehabilitation studies. Moreover, since computerized tests are hard-coded, their administration is less sensitive to the identity of the experimenter and environmental variability.

The sensitivity of computer-based approaches was evident in recent studies (Bonato et al., 2010, 2012, 2013) in which the presentation of brief lateralized stimuli was combined with resource-demanding tasks, two methodological characteristics which maximize the possibility to detect contralesional omissions. Post-acute (1–3 months from stroke) right-hemisphere damaged patients were tested in three conditions. In the single-task condition only the position of the target(s) had to be verbally reported. In the two dual-tasks, while monitoring for target(s) appearance, patients also had to perform a concurrent task. In the visual dual-task they had to report a centrally presented letter, while in the auditory dual-task they had to count at steps of two from an auditorily presented number. Both extinction rate for bilateral targets and omission rate for unilateral contralesional targets dramatically increased under dual-task conditions, even in patients who were normal according to clinical standards for neglect such as the Behavioral Inattention Test (BIT, Wilson et al., 1987). A patient who was followed-up for several months after discharge and showed deficits during the dual-task conditions, similarly showed severe deficits in attention-demanding everyday life contexts (Bonato et al., 2012) despite normal performance at the BIT. Another sensitive approach has been proposed by Deouell et al. (2005) using the Starry Night Test (SNT). In the SNT, relatively brief targets can appear in many spatial positions on a computer screen. Spatial uncertainty plausibly deploys attentional monitoring resources and hampers the implementation of compensatory strategies. Moreover, in the SNT, the presence of flickering distracters across the display does not allow patients to respond as soon as something appears on the screen (pop-out) but forces them to identify the target before responding. Deouell et al. demonstrated a higher sensitivity in the SNT compared to the BIT at the individual level, and described in detail the deficits shown in everyday life by a patient whose neglect was only evident in the SNT (see also Erez et al., 2009). Moreover, some patients with normal behavior by the BIT at the early stage, who showed slow reaction times on the left in the SNT, achieved more symmetric RTs after a period of recovery (Sacher et al., 2004).

The Dual-Task, and the SNT paradigms were described in some detail above to illustrate the principle based on our own experience, and not in order to endorse those specific tests over others. Several other computerized tests were shown to unveil unilateral neglect (see Bonato, 2012 for review). These tests include variants of visual perimetry (Müller-Oehring et al., 2003; Nijboer et al., 2011), variants of the classic Posner-like detection tasks which can provide RTs measures for contralesional vs. ipsilesional hemispace (Bartolomeo, 1997; Nijboer et al., 2008; Rengachary et al., 2009), feature and conjunction search tasks (Erez et al., 2009), as well as tasks manipulating load (e.g., Russell et al., 2004, in press; Buxbaum et al., 2008, 2012; Dawson et al., 2008; Bellgrove et al., 2013; van Kessel, et al., 2010, 2013). These computer-based tasks are typically well tolerated by patients in the post-acute and chronic phases after a stroke, when tasks’ differential sensitivity with respect to PnP tests is maximal. Dealing with a computer is, typically, relatively easier for those patients without neglect at PnP tests.

Although no study to date compared the sensitivity of these heterogeneous computer-based tests, most if not all demonstrated improved sensitivity to residual deficits with respect to standard clinical tests. Moreover, these tasks can be more easily tailored to recruit cognitive resources close to those adopted in everyday life, reducing the gap between everyday life and neuropsychological testing. Given that the average performance in PnP tests is frequently dissociated from performance in everyday life (Hasegawa et al., 2011), it has been considered mandatory to resort to independent measures to quantify impairments in ADL (Azouvi et al., 2002). While the final aim of rehabilitation is to increase the independence of the patients, the scales adopted to measure everyday performance such as FIM, Barthel, and Bergego only allow quantifying disability in “easy” tasks such as eating or dressing, but do not appear to be sensitive enough to detect either subtle neglect in complex settings or small differential improvements in everyday life activities. Additionally, they do not discern whether performance is impaired due to contralesional motor, intentional, or attentional problems or to a combination of those deficits (but see Eschenbeck et al., 2010 for neglect-specific ADL assessment). It seems that, somewhat paradoxically, in computer-based tasks allowing less compensatory strategies, the dissociation between daily life and testing performance which often characterizes the chronic phase is reduced relative to the PnP tests. By virtue of their added level of complexity and flexibility, computerized tasks have the potential to simulate the performance of patients in everyday life by reproducing the cognitive demands everyday life requires. After their discharge from the hospital, some patients performing normally at PnP tests but showing impairments in computer-based tasks also show severe impairments in everyday life (Deouell et al., 2005; Bonato et al., 2012). Furthermore, performance at computer-based tasks may correlate with ADL performance (Erez et al., 2009) and with a real world task (Buxbaum et al., 2012). Notably, the performance of older drivers in a computer-based visual dual-task (UFOV) is highly predictive of car crash problems (Ball et al., 1993, see also the case report in Deouell et al., 2005). Thus, computer-based approaches may eventually help clinicians in evaluating and predicting individual performance in everyday demanding situations. A first step for future research would be to further establish the ecological validity of these new tests and their correlation with the level of disability and handicap.

Despite the advantages of the computerized tests, we do not suggest that time honored PnP should be completely discarded. Over the years, many such tests have been developed, likely capturing non-overlapping aspects of neglect. Although patients’ individual performance often dissociates according to the task and spatial domain under investigation (Halligan and Marshall, 1991; Azouvi et al., 2002; Buxbaum et al., 2004; Sacher et al., 2004; Sarri et al., 2009) computerized tests tapping all the multiple components of neglect are still missing. Computerized tests also have practical limitations, as normative data are not present for all tests and they require dedicated hardware and software not always available at the clinical setting, although this is likely to become less of a problem in the (near) future.

To conclude, we argue that the inclusion of quantitative, standardized, computerized tests, recording a continuous measure like RT, as well as accuracy, and allowing to increase task difficulty to reduce the effect of compensatory strategies, have major advantages over traditional PnP tests in the context of evaluating spontaneous recovery and the effects of rehabilitation interventions. The sensitivity of these methods have the potential of detecting ecologically meaningful improvements in patients’ performance, which are missed using traditional PnP tests. More effort needs to be done in devising such tests that will tap into various aspects of UN, correlated with the patients’ handicap. Like jewelers weighing precious stones, neuropsychologists need to adopt sensitive scales before and after the implementation of a valuable technique for neglect rehabilitation.

## Author Note

To obtain copies of the dual-task or of the SNT task, contact mariobonato@hotmail.com or msleon@mscc.huji.ac.il.
